# Sensitivity of viscoelastic characterization in multi-harmonic atomic force microscopy†

**DOI:** 10.1039/d2sm00482h

**Published:** 2022-11-08

**Authors:** Abhilash Chandrashekar, Arthur Givois, Pierpaolo Belardinelli, Casper L. Penning, Alejandro M. Aragón, Urs Staufer, Farbod Alijani

**Affiliations:** Faculty of Mechanical, Maritime and Materials Engineering, Delft University of Technology Mekelweg 2 2628 CD Delft The Netherlands arthur.givois@utc.fr f.alijani@tudelft.nl; DICEA, Polytechnic University of Marche Ancona Italy

## Abstract

Quantifying the nanomechanical properties of soft-matter using multi-frequency atomic force microscopy (AFM) is crucial for studying the performance of polymers, ultra-thin coatings, and biological systems. Such characterization processes often make use of cantilever's spectral components to discern nanomechanical properties within a multi-parameter optimization problem. This could inadvertently lead to an over-determined parameter estimation with no clear relation between the identified parameters and their influence on the experimental data. In this work, we explore the sensitivity of viscoelastic characterization in polymeric samples to the experimental observables of multi-frequency intermodulation AFM. By performing simulations and experiments we show that surface viscoelasticity has negligible effect on the experimental data and can lead to inconsistent and often non-physical identified parameters. Our analysis reveals that this lack of influence of the surface parameters relates to a vanishing gradient and non-convexity while minimizing the objective function. By removing the surface dependency from the model, we show that the characterization of bulk properties can be achieved with ease and without any ambiguity. Our work sheds light on the sensitivity issues that can be faced when optimizing for a large number of parameters and observables in AFM operation, and calls for the development of new viscoelastic models at the nanoscale and improved computational methodologies for nanoscale mapping of viscoelasticity using AFM.

## Abstract 2

This study discusses the sensitivity of viscoelastic characterization to multi-frequency atomic force microscopy observables.

## Graphical abstract

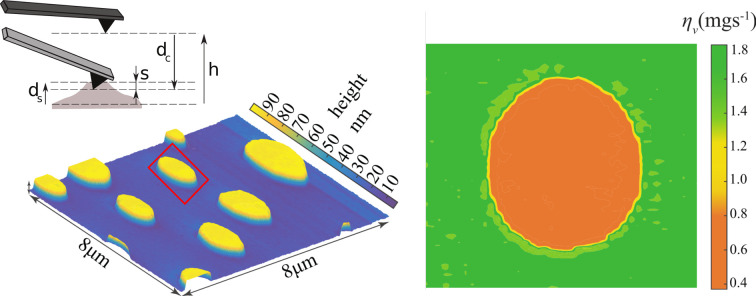

## 1 Introduction

Viscoelastic characterization of soft-matter at the nanoscale is important for understanding cell membrane functioning,^1–4^ developing innovative materials in polymer science,^5–7^ and for advancing nanolithography.^8,9^ In this regard, dynamic atomic force microscopy (AFM) has emerged as an indispensable tool for characterizing nanomechanical properties of soft matter, offering diverse operating conditions under which a wide variety of samples can be probed with gentle forces.^10,11^

Dynamic AFM imaging offers multiple observable channels in the form of higher harmonics, modal amplitude, and phase contrast signals to map nanomechanical properties. Among multi-harmonic AFM techniques, the emergence of bi-modal and intermodulation AFM (IM-AFM) has led to a drastic increase in the number of experimental observables and a consequent advancement in our understanding of material properties at the nanoscale. In particular, IM-AFM extends the concept of multi-frequency observables by providing a fast and convenient method to measure a set of frequency components in a narrow frequency band centered around the fundamental resonance of the AFM cantilever.^12,13^ These frequency components directly benefit from the mechanical resonance gain of the first mode and can be easily converted to tip-sample force quadratures, which are in turn linked to the conservative and dissipative interactions with a sample.^13,14^

Despite the advancements in AFM instrumentation and the abundance of viscoelastic models at hand,^15–20^ a consistent and robust estimation of viscoelasticity using AFM has remained a challenge.^4^ This is mainly due to the fact that the compositional contrast of AFM images depend on several nanomechanical properties including elasticity, surface relaxation, and adhesion. Untangling these effects from one another requires setting up an optimization problem, where a large parameter space has to be searched to minimize the error between the simulations from a model and experimental data. But, similar to any optimization problem, the insensitivity of the model parameters with respect to the measurement data on one side, and the non-convexity of the objective function on the other side, can lead to non-unique and often non-physical estimation of parameters. Therefore, knowledge about the sensitivity of the model parameters to AFM observable channels is of paramount importance to extract consistent and reliable viscoelastic properties in dynamic AFM applications.

In this article we discuss the sensitivity issues that can arise when characterizing viscoelasticity using multi-frequency IM-AFM. We perform measurements on a polymer blend made of stiff Polystyrene (PS) and soft Low-Density-Polyethylene (LDPE), and use a moving surface model^19,21^ to extract the bulk and the surface viscoelasticity. The estimation of viscoelastic properties is achieved by matching the experimental spectral components of tip-sample force to the ones predicted by a computational model *via* an optimization procedure. To ascertain the sensitivity of the model parameters on the physical observables, we perform a comprehensive comparison involving both local and global optimization techniques, and reveal a lack of sensitivity of surface motion to the experimental data obtained from IM-AFM. We show that the issue of insensitivity manifests itself during the optimization of the objective function by means of a vanishing gradient with respect to the surface parameters. To overcome this problem, we introduce a simple model, neglecting surface motion, which leads to statistically consistent and robust identification of bulk viscoelastic parameters. This work thus provides a general framework that can be used for investigating the reliability of similar viscoelastic models used for nanomechanical characterization in multi-frequency AFM applications.

## 2 Experimental results

**Fig. 1:**
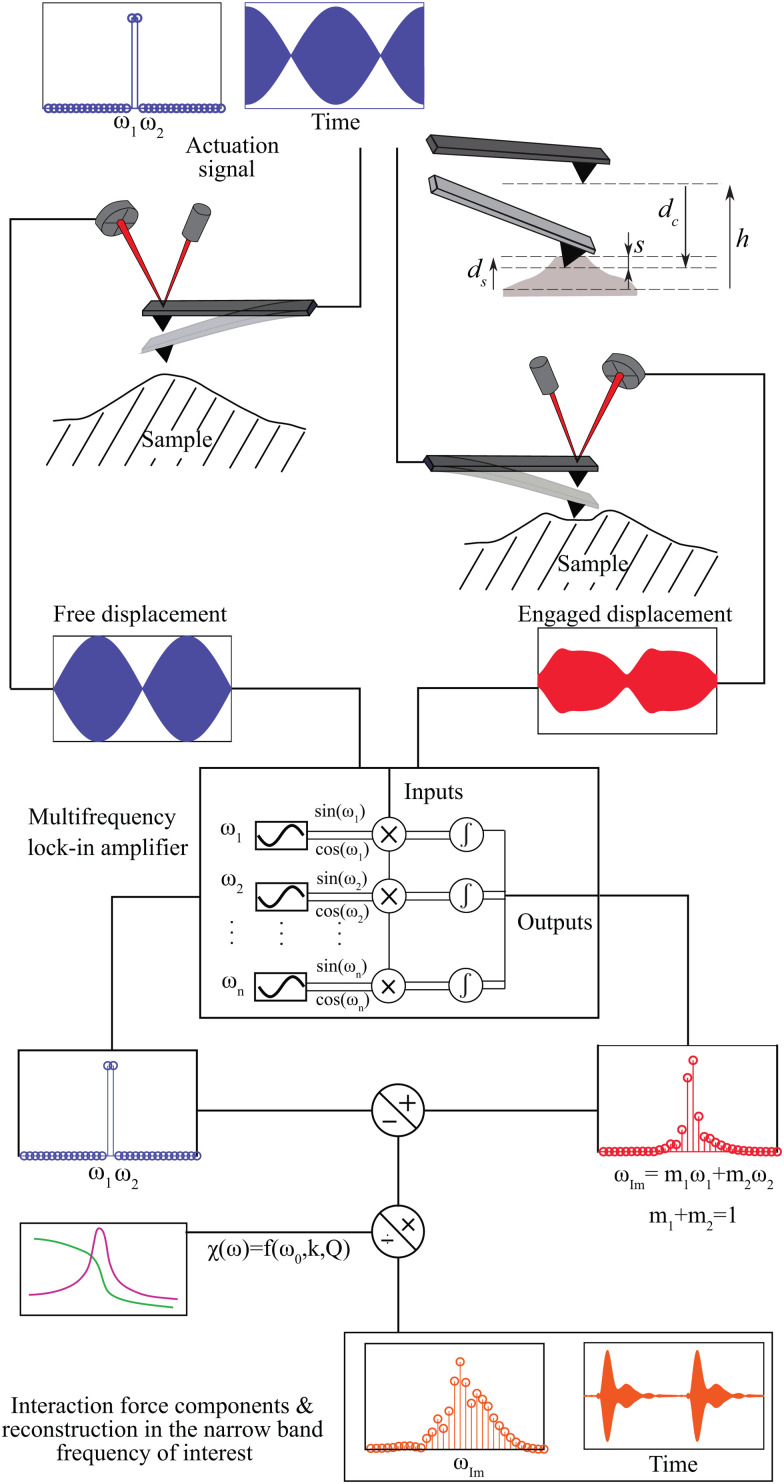
Schematic of the working principle of the IM-AFM. The cantilever is driven with a signal comprising two close frequencies *ω*_1_ and *ω*_2_, centered around its first resonance frequency. The intermodulation distortion caused by the nonlinear tip-sample interaction creates frequency comb at commensurate frequencies *ω*_IM_ = *m*_1_*ω*_1_ + *m*_2_*ω*_2_, with *m*_1_,*m*_2_ ∈ *Z*. The linear transfer function of the cantilever *χ*(*ω*) is measured *via* thermal calibration, and the amplitudes and phases of these intermodulation products are captured using a multi-lock-in amplifier. Here, *d*_c_ and *d*_s_ denote the tip cantilever and surface vertical displacements and *h* corresponds to the working distance between tip and sample. Finally, *s* = *h* + *d*_c_ − *d*_s_ represents the tip-sample distance.

We perform our experiments with a commercial AFM (JPK nanowizard 4) and use a multi-lock-in amplifier (Intermodulation products AB) to measure and analyse the frequency components resulting from the tip-sample interaction. A rectangular Silicon cantilever (Tap190Al-G, BudgetSensors) probes the viscoelastic response of a polymer blend made up of PS-LDPE materials. The stiffness of the cantilever (*k* = 26.70 N m^−1^), its resonance frequency (*f*_0_ = 153.9 kHz) and the quality factor (*Q* = 596) are determined using the thermal calibration method.^22^ A schematic of the intermodulation AFM setup is shown in Fig. 1. The cantilever is excited with two frequencies centered around its fundamental mode of vibration. The interaction of the cantilever with the sample, under the influence of nonlinear surface forces, generates frequency combs that are measured using the lock-in amplifier. In particular, the amplitude and phase of the combs are used as experimental inputs for the viscoelastic identification procedure. Details of IM-AFM operation and processing of the experimental data can be found in ref. 12, 13, 21, 23, we summarize the essential operations in Section S1 of the ESI.†

**Fig. 2:**
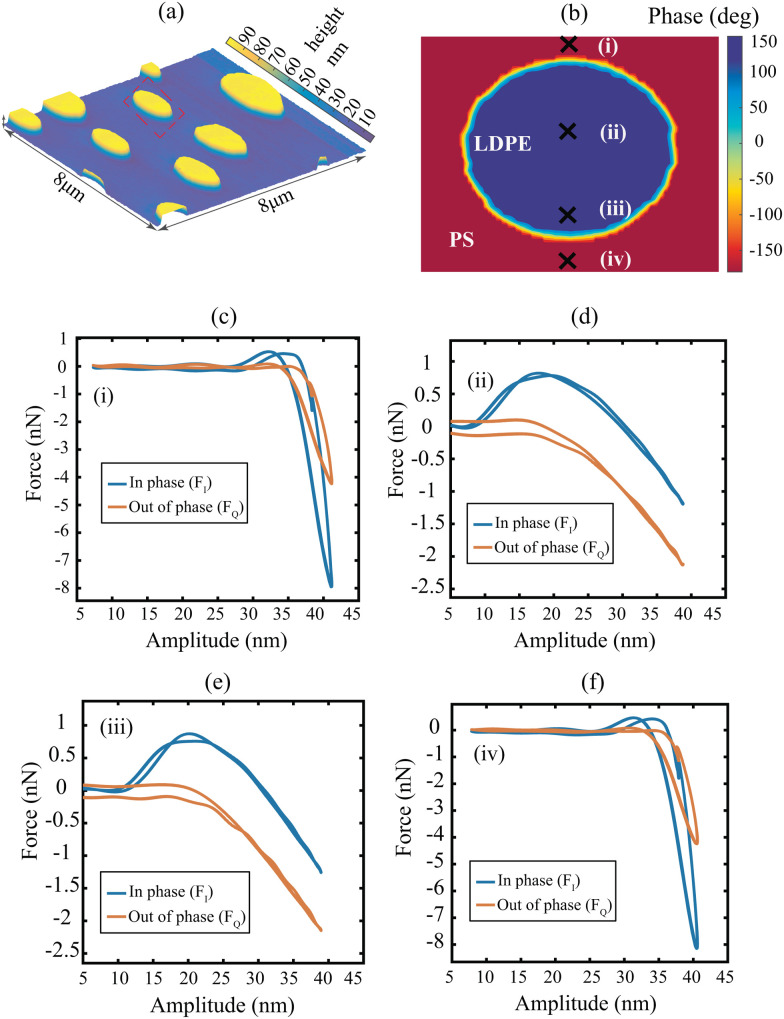
Experimental measurements performed on the PS-LDPE polymer blend. (a) Amplitude image at the second drive frequency (*ω*_2_), which is part of the 32 different image pairs captured during the scanning operation. (b) Phase image at the second drive frequency. The image shows an island of LDPE within the PS matrix (red dashed box in Fig. 2(a)). The points of measurements are indicated with black crosses. (c–f) Experimental force quadratures obtained at the pixels marked by black crosses in the phase image. The quadratures in subfigures (c–f) are obtained on PS material, whereas the quadratures in sub figures (d–e) are obtained on LDPE material.

The experiments performed on the PS-LDPE polymer blend are reported in Fig. 2. Fig. 2(a and b) depict the amplitude and phase images at the second drive frequency *ω*_2_. The phase image is presented for one specific LDPE island surrounded by PS matrix. In total 32 amplitude and phase intermodulation components are used to reconstruct the tip-sample interaction in the narrow frequency band around the fundamental resonance. Furthermore, the frequency components are used to calculate the tip-sample force quadratures, which represent the time averaged interaction force that the cantilever experiences in one oscillation cycle (see Fig. 2(c–f) for both PS and LDPE). The force quadratures are a local measure of material properties since they are calculated for every pixel of the AFM image; they provide information about the conservative and dissipative contributions of the interaction force between the tip and the sample. For instance, the in-phase quadratures provide information about the amount of adhesive (positive part) and repulsive (negative part) forces at the measured pixels.^21^

## 3 Modelling tip-sample interaction

In order to probe the viscoelastic response of the sample and interpret the in-phase and out-of-phase quadrature information quantitatively, we begin by describing the dynamics of the AFM cantilever using the following simple model:^24,25^1
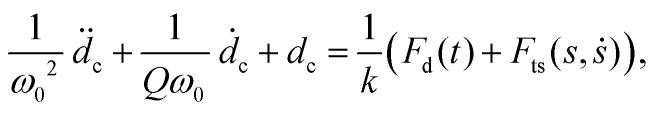
where *d*_c_ describes the total deflection of the cantilever from its equilibrium, *ω*_0_ = 2π*f*_0_ denotes its resonance frequency, *k* represents the stiffness of the cantilever, *t* denotes the time and *F*_d_ is the excitation force. The above equation couples to the sample through the nonlinear tip-surface force2
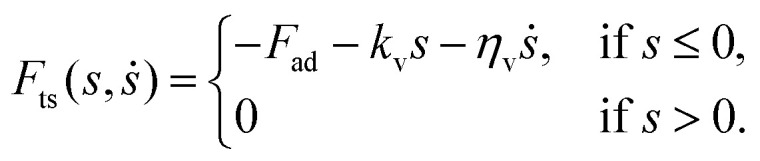
Here, the piecewise linear (PWL) model assumes *F*_ts_ to be function of the indentation (*s*) and the rate of indentation (*ṡ*). In eqn (2), the tip-sample interaction comprises of an adhesion force represented by *F*_ad_, a repulsive force due to surface indentation governed by the bulk sample stiffness *k*_v_, and finally, a viscous force due to material flow upon indentation governed by the coefficient *η*_v_. It must be noted that the PWL model preserves an essential feature of the interaction that is well-known in AFM, which is the presence of large force gradient localized near the point of contact, *i.e* at *s* = 0. This rapid change of force is responsible for the jump-to-contact and pull-off hysteresis seen in nearly all quasi-static force curves in AFM. However, in dynamic AFM, the oscillation amplitude is typically much larger than the range of this localized interaction. Hence, we approximate this region of large interaction gradient as an adhesion force that instantly turns on and off when crossing the point of contact, whose magnitude is counterbalanced by the contribution of the velocity-dependent term *η*_v_*ṡ*.

We then couple the cantilever dynamics with a moving surface model^19,21^ to account for the motion of the sample interacting with the tip

3 *η*_s_*ḋ*_s_ + *k*_s_*d*_s_ = −*F*_ts_(*s*,*ṡ*).

here, the stiffness and viscosity of the sample surface are *k*_s_ and *η*_s_, respectively. The instantaneous surface motion is related to the cantilever oscillation through the relation *s* = *h* + *d*_c_ − *d*_s_, where *h* is the height between the apex of the cantilever tip and the unperturbed sample position as shown in Fig. 1. Unlike classical contact mechanics models, our model implicitly accounts for the effects of tip radius and indentation rate *via* the effective bulk stiffness *k*_v_ and the surface stiffness *k*_s_ parameters.

The tip-sample interaction process as described by eqn (1)–(3) introduces a large set of unknown parameters that shall be extracted from the intermodulation components. However, few of them, namely *ω*_0_, *Q*, and *k* are obtained directly from thermal calibration.^26^ This reduces the unknown set of parameters that needs to be identified to ***P*** = {*F*_ad_, *k*_v_, *η*_v_, *k*_s_, *η*_s_, *h*}. At this stage, the optimization problem is written as:

4 find min_***P***∈ℝ^6^_*f*(***P***)

with *f*(***P***) the objective function defined as:^13,27,28^5
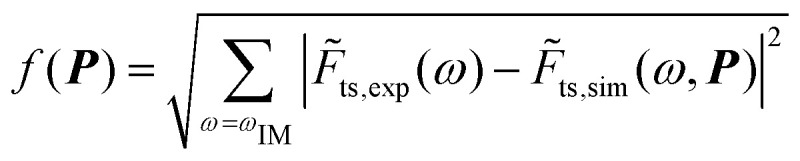
where *F̃*_ts,sim_ and *F̃*_ts,exp_ denote the complex spectral components of the simulated and experimental interaction force at the intermodulation frequencies *ω*_IM_, respectively.

## 4 Linking viscoelasticity to intermodulation components

We start the identification by analyzing the two pixels denoted by (i) and (iii) in Fig. 2(b). These pixels belong to the PS and the LDPE material, respectively. The optimization of the model parameters is carried out using the Levenberg–Marquardt algorithm since it has strong convergence properties and robustness against numerical inconsistencies.^29^ We note that the minima obtained by the optimizer are largely dependent on the initial points (IP) chosen for the unknown parameter set ***P***. Thus, several initial starting configurations are tested for the identification procedure; these are selected based on values previously reported in the literature^30–33^ (see Section 3 in ESI† for additional details).

**Fig. 3:**
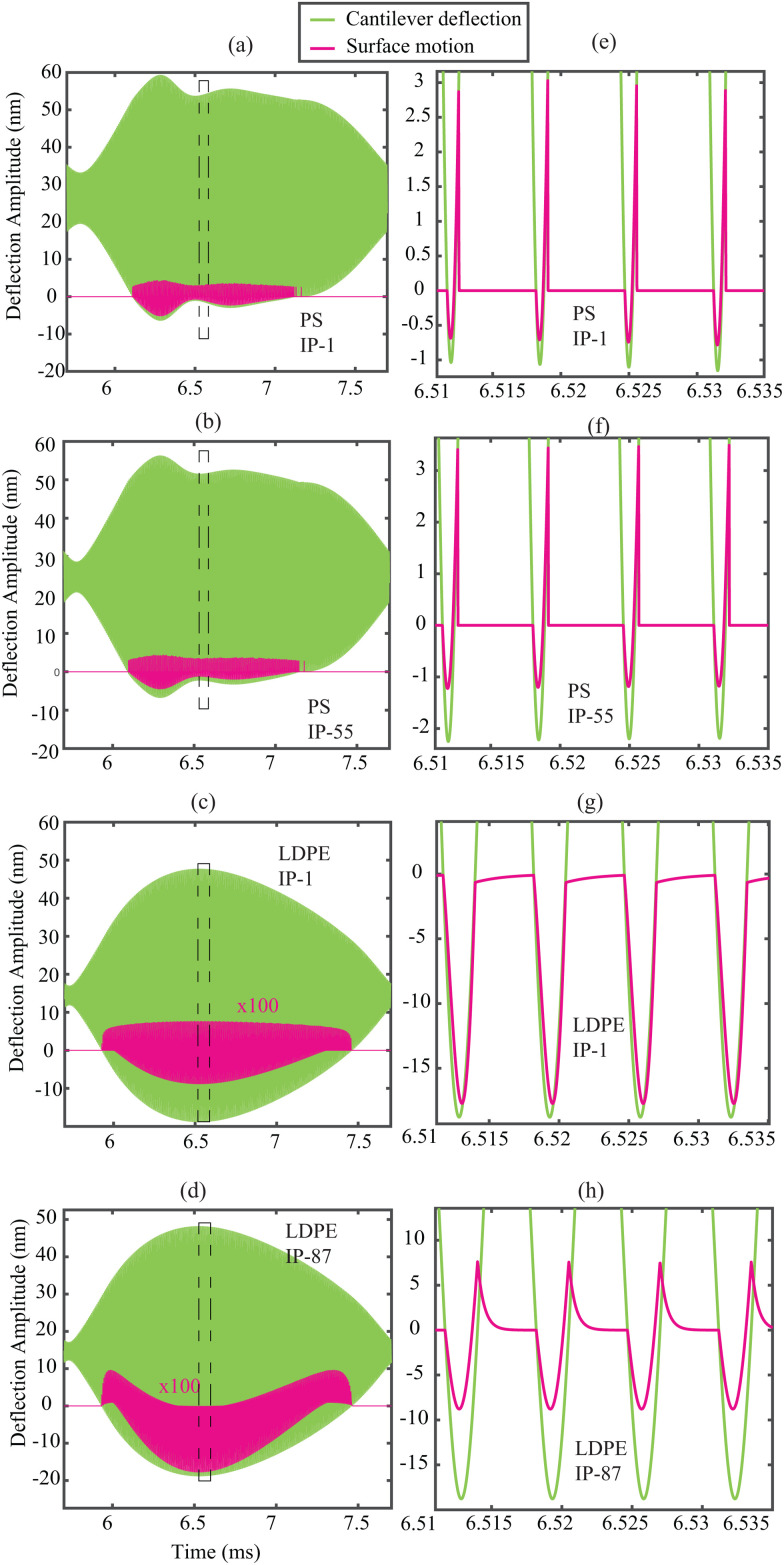
Simulations of the cantilever (green) and sample (pink) surface dynamics based on the results provided in Table 1. (a and b) Simulated results for PS material with parameter values taken from IPs 1 and 55, respectively. (c and d) Simulated results for LDPE material with parameter values taken from IPs 1 and 87. (e–h) A close up visualization of the surface dynamics is reported in (a–d).

Table 1 summarises the identified model parameters and the corresponding errors between the simulation and the experimental counterparts for several different IPs on pixels (i) and (iii). Here, we note that the surface stiffness (*k*_s_) and damping (*η*_s_) of LDPE are much higher than PS matrix. This qualitative and counter-intuitive result can be explained considering that LDPE has a smaller Young's modulus than PS and thus is prone to a larger penetration and contact area, resulting in a larger *k*_s_. Nonetheless, we observe that the estimated values of the surface parameters vary by several order of magnitudes without significant change of the objective function, which raises questions about the reliability of the identification of surface parameters. Furthermore, we make a qualitative visual comparison in Fig. 3(a) and (b) by reconstructing the cantilever motion (green) and the surface motion (pink) and highlight that the signals look identical, even though the identified parameter values exhibit large differences. Additionally, in Fig. 3(c) and (d) the surface motion in case of LDPE is strongly dependent on the choice of IPs and consequently leads to different parameter value estimations. Contrary to the popular notion which considers that surface and tip displacements are identical during contact, the moving surface model presented in Section 3 allows to characterize different surface motion amplitudes. In particular, the magnitude of the surface displacement in case of soft LDPE is much smaller compared to the stiff PS material.

**Table 1:** Extracted results from a large set of local minimization routines using Levenberg–Marquardt algorithm, using the model which includes surface motion and the grid of initial points (IPs) defined in Table S3.5 of ESI. The initial points are ranked according to the best results, defined here as the lowest errors/highest *R*^2^

Initial point	Pixel (i)-PS			Pixel (iii)-LDPE		
	IP 1	IP 55	IP 99	IP 1	IP 22	IP 87
*F*_ad_ (nN)	30.5	31.6	41.6	7.08	7.12	7.13
*k*_v_ (N m^−1^)	94.9	43.2	89.5	0.848	0.854	0.860
*η*_v_ (mg s^−1^)	15.5	7.33	6.60	0.520	0.521	0.521
*k*_s_ (N m^−1^)	18.8	16.8	11.8	123.8	239.3	28.4
*η*_s_ (mg s^−1^)	0.0552	0.00884	0.993	57.2	0.0594	62.0
*h* (nm)	26.35	24.69	24.11	14.43	14.69	14.67
Final *E* (nN)	0.511	0.537	0.579	0.193	0.194	0.194
*R* ^2^	0.961	0.957	0.950	0.979	0.979	0.979

**Fig. 4:**
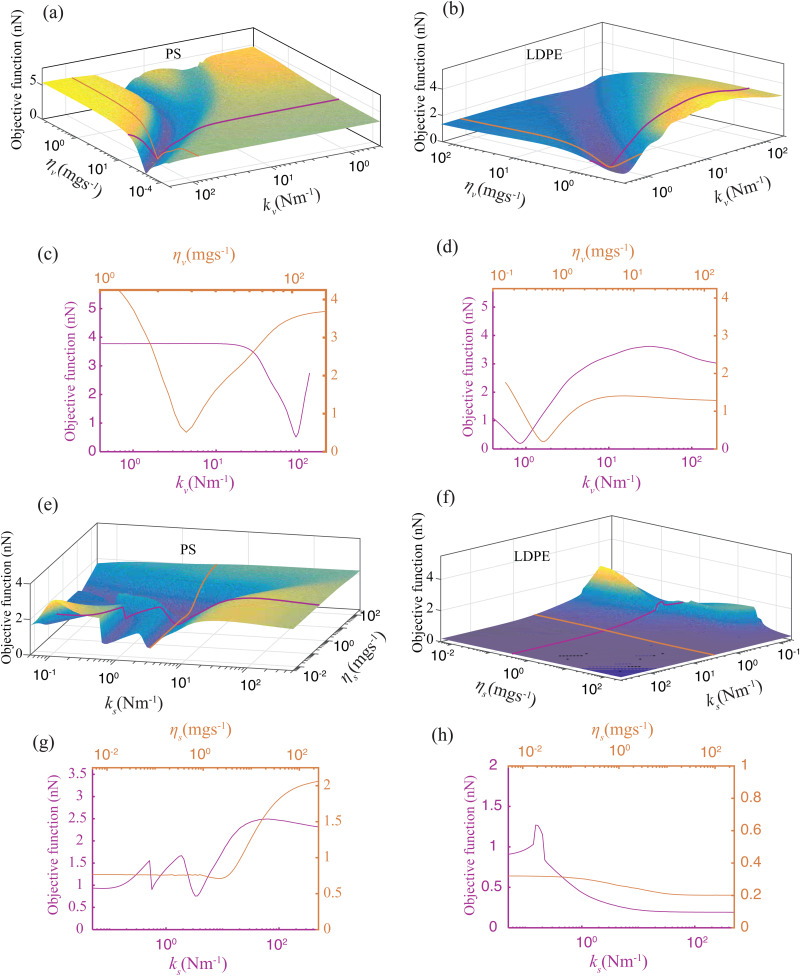
Variation of the objective function in a 2-dimensional parameter space comprising ((*k*_s_,*η*_s_) or (*k*_v_,*η*_v_)), with the other parameters fixed in accordance with the best results found from the local minimization routine. (a–d) Visualizing the landscape of the minimization objective as a function of *k*_v_ and *η*_v_ for PS and LDPE material obtained at pixel (i) and (iii) of Fig. 2(b). The purple and orange lines indicate a 2D cross-sectional view of the objective function. (a–d) Visualizing the landscape of the minimization objective as a function of *k*_s_ and *η*_s_ for PS and LDPE material obtained at pixel (i) and (iii) of Fig. 2(b). Purple and orange lines indicate 2D cross-sectional views of the objective function.

We relate the above remarks to possible insensitivity of the objective function towards certain model parameters and the presence of multiple local minima, which indicates that the objective function is non-convex. To elaborate on these issues, we analyze the topography of the objective function in the space of model parameters on large variable ranges. We note that the objective function includes 6 parameters, out of which *F*_ad_ and *h* show consistent convergence. Hence, we limit our analysis to the bulk and surface viscoelastic parameters governed by *k*_v_, *η*_v_, *k*_s_, and *η*_s_. This is showcased in Fig. 4, where the topographies of the objective function are obtained by sweeping across the viscoelastic parameters for both PS and LDPE material at pixels (i) and (iii), respectively. In each sub-figure, the four non-varied parameters are chosen as those of IP 1 in Table 1. Interestingly, we note that Fig. 4(a) and (b) exhibit a valley in which a single optimum solution is found. This is further highlighted in the 2D cross sections shown as Fig. 4(c) and (d), confirming the strong dependency of parameters *k*_v_ and *η*_v_ on the experimental observables. Contrary to this, the landscape of Fig. 4(e) and (f) highlight multiple local minima (in the case of pixel (i) in Fig. 4(e)) and a flat topography (for pixel (iii), in Fig. 4(f)). A flat landscape indicates the insensitivity of the objective function to the surface parameters (*k*_s_,*η*_s_) in this region of the parameter space. This behaviour is also reflected in the large spread of values reported in Table 1.

In order to verify that these issues do not stem from the local optimizer used in our simulations, we also employ a heuristic global optimization technique in pursuit of a global solution in the parameter space. We create synthetic data sets with known optima to analyse how the global optimizer performs (for details see Section 2B in ESI†). We note that the use of this global optimization strategy also does not lead to a robust identification of the surface parameters. Indeed, a wide range of parameter values which differ from several orders of magnitude allows for reconstructing the original cantilever motion, and large differences in the surface viscoelastic parameter *η*_s_ do not affect the objective function. Upon closer inspection of results, we noticed a trend for synthetic data sets with good solution convergence, where the bulk parameters of the model, namely *k*_v_, *η*_v_, tends to the original optimum (for details see Table S2.2 in Section 2B of ESI†). This is in accordance with our hypothesis regarding the insensitivity of surface viscoelastic parameters on the experimental observables. Therefore, fine-tuning of the global optimization parameter space is effective in determining bulk viscoelastic parameters. Nevertheless, isolation of non-physical solutions as outliers is computationally expensive when aiming for fast parameter estimation. For this reason we explore an alternative local optimization route paired with an initial point selection procedure in the following section.

### Estimating bulk viscoelasticity in the absence of surface motion

In order to overcome the aforementioned limitations as well as to improve the computational efficiency for the parameter estimation procedure, we neglect the surface dynamics of the sample and reduce the unknown parameter set to ***P̄*** = {*F*_ad_,*k*_v_,*η*_v_,*h*}. This assumption holds in particular for the case where the surface displacement is much smaller than tip displacement (|*d*_s_| ≪ |*d*_c_ + *h*|). This hypothesis is valid for the LDPE (softer) material according to the displacement signals shown in Fig. 3. It must be noted that this reduced set is still descriptive of the nanomechanical mapping of polymer blends and coherent with several well-established formulations, *e.g.*, Derjaguin–Muller–Toporov (DMT)-Kelvin–Voigt,^30^ 3D Kelvin–Voigt,^34^ and DMT–Garcia.^35^

We begin by repeating the quantitative analysis at pixels (i) and (iii) of Fig. 2, once again applying the Levenberg–Marquardt algorithm. In this procedure we use a grid of 3^4^ IPs, by defining three values for the four free parameters of the model. This choice of three values is motivated by a compromise between a wide range of parameter exploration and a reasonable simulation duration. These parameter values include in particular at least one order of magnitude for the viscoelastic properties (for details see Section S3B in ESI†). Furthermore, the three values of the probe height *h* can be framed from the force quadrature profiles and from onsets of repulsive forces (for details see Section S2C in ESI†). We then perform a gradient-based optimization for each combination of parameters in the parameter space and conduct statistical analysis by obtaining the Gaussian distribution profiles of the identified parameters (for more details see Section S3B in ESI†). Interestingly, for most of the IPs the optimizer converges towards an admissible physical solution.

Based on this statistical analysis we extract a set of three initial points for performing the parameter identification at all pixels of the entire AFM scan. The first two sets of IPs are derived from the mean values of the Gaussian distribution for both the PS and LDPE material. Indeed, these mean values lead to the lowest errors at pixels (i) and (iii). As for the third set, an IP is chosen which can lead to a set of identified parameter within a specific confidence interval for both the PS and LDPE material. The reasoning for choosing such an IP is rooted in our optimization procedure where, we assume that pixels belonging to the same material have similar objective function topology. This assumption may not hold true at the junctions where the two materials blend. Hence, having a third IP that could identify the parameters of both PS and LDPE material within a certain confidence interval is crucial to avoid non-physical parameter estimation (for details see Section 3.2 of ESI†). Finally, among the three optimization run at each pixel, we retain the parameters of the best fit (*i.e.* the lowest error) as the identified model parameters.

**Fig. 5:**
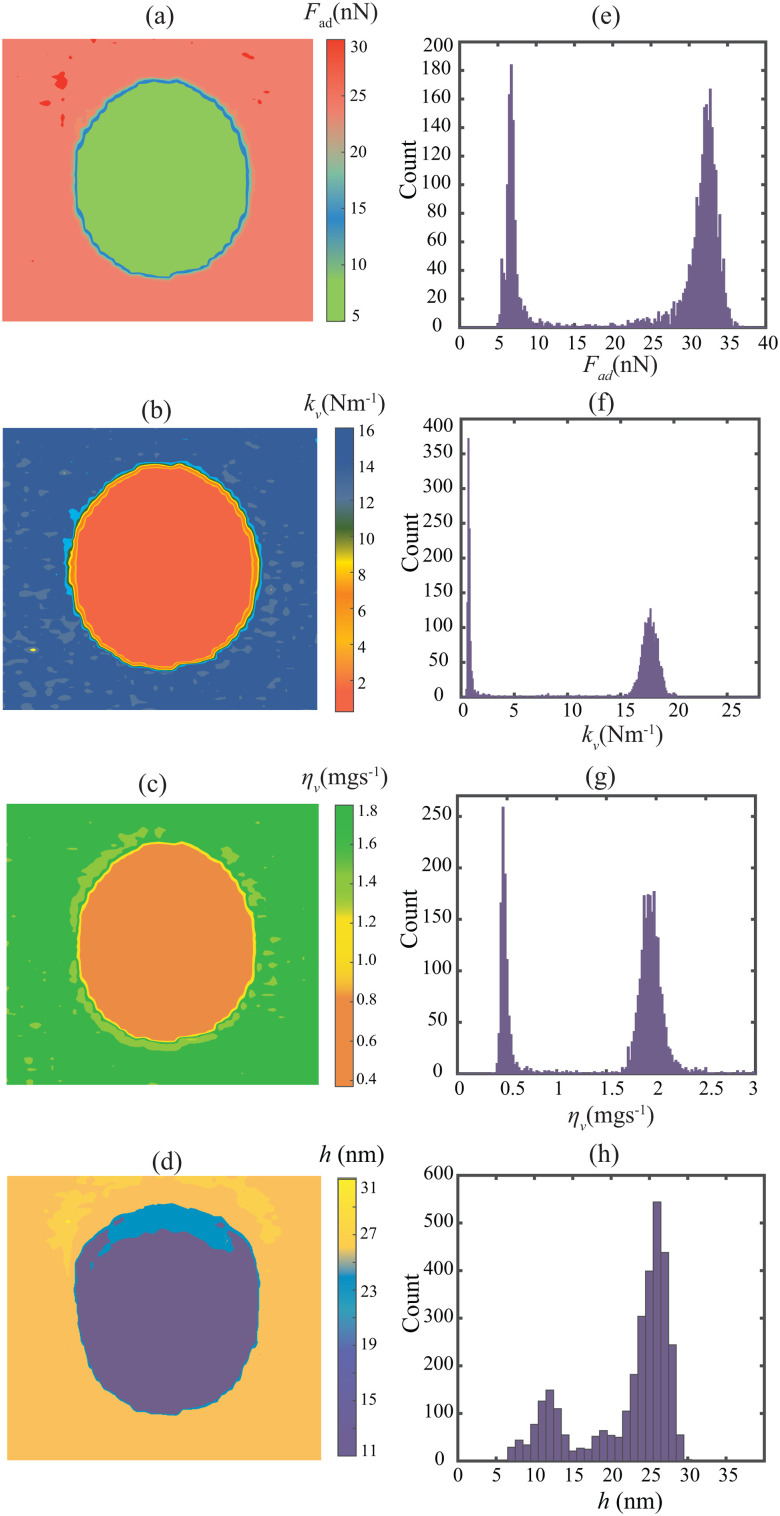
Estimated properties of the PS-LDPE sample obtained using the model without surface motion and the initial points selection procedure described in this work. The maps are of dimensions 2.5 μm × 2.5 μm. Left: Parameter maps of (a) adhesion force, (b) contact force stiffness, (c) contact force viscosity, (d) probe height. Right: Histogram distribution of the respective parameters (e–h).

Fig. 5 shows the identified parameter values for the PS-LDPE polymer blend. It highlights a clear distinction between the identified bulk parameters *F*_ad_, *k*_v_, and *η*_v_ between the island of LDPE and the surrounding PS matrix. This can be seen in the observed compositional contrast in the colored figures. Additionally, the histogram displayed on the right side of the figure highlights clear separated Gaussian profiles for each of the parameters. The estimated values lie within a 95% confidence interval for the entire image, as Table 2 shows. Moreover, we remark that our identified values are in line with those previously reported in the literature^30–33^ and align with the expected physical behaviour of the two polymers, *i.e.* (*F*_a,PS_ > *F*_a,LDPE_, *k*_v,PS_ > *k*_v,LDPE_ and *η*_v,PS_ > *η*_v,LDPE_). Our analysis suggests that intermodulation frequency components have a direct correlation with the bulk properties of the sample and the interaction force function can be robustly characterized.

**Table 2:** Identified parameters resulting from the Gaussian fits, made from the material properties estimated at all pixels plotted in Fig. 5. The uncertainties are estimated with a 95% confidence interval

	PS	LDPE
*F*_ad_ (nN)	31.49 ± 0.12	6.960 ± 0.076
*k*_v_ (N m^−1^)	17.31 ± 0.09	0.819 ± 0.020
*η*_v_ (mg s^−1^)	1.951 ± 0.007	0.492 ± 0.005
*h* (nm)	26.71 ± 0.02	12.86 ± 0.021

## 5 Conclusions

In summary, we studied the dependency of viscoelastic response of polymeric samples to multi-frequency IM-AFM. We discussed the sensitivity issues that can be faced when minimizing the error between IM-AFM spectral components and a tip-sample force model with surface dynamics, and confirmed that insensitivity of surface viscoelasticity to experimental observables could lead to non-physical parameter estimations. We attribute this finding to the non-convexity and flat landscapes of the objective function with respect to the sample's surface viscoelastic parameters. This was further reinforced with numerical simulations that used both gradient-based and heuristic global optimization techniques. We remedy this issue with a simplified model that only accounts for the bulk viscoelastic parameters and by implementing an initial point selection procedure that searches a large parameter space to estimate model unknowns with ease. This new framework results in consistent identification of viscoelastic parameters that are in good agreement with previously reported values. However, in order to take full advantage of the vast amount of multi-frequency observables, a more accurate and sensitive viscoelastic tip-surface model is needed,^15,20,32^ and computational developments to speed up the optimization process are required. Finally, given the growing interest in developing multi-parametric techniques in multi-frequency AFM, we believe that the techniques showcased in this work can be useful in providing guidance to future investigations that are aimed at studying soft, adhesive and viscoelastic surfaces of samples.

## Data availability

The authors declare that all the data in this manuscript are available upon reasonable request.

## Author contributions

A. C., C. P., and F. A. conceived the experiments. A. C. and A. G. prepared the samples and conducted the experiments. A. C., A. G., P. B., and F. A. conceived the simulations. A. G. and C. P. conducted the simulations. A. C., A. G., P. B., C. P., A. A. and F. A. did data analysis and interpretation. U. S. and F. A. supervised the project. All the authors jointly wrote the article with main contribution from A. C. and A. G. All authors discussed the results and commented on the article.

## Conflicts of interest

There are no conflicts to declare.

## Supplementary Material

SM-018-D2SM00482H-s001
