# Supplementary material for: Sensitivity of viscoelastic characterization in multi-harmonic atomic force microscopy
Source: Soft Matter. 2022 Nov 8;18(46):8748–55. doi: 10.1039/d2sm00482h (PMC9709660; doi:10.1039/d2sm00482h)
Supplement: SM-018-D2SM00482H-s001 [file SM-018-D2SM00482H-s001.pdf]

Electronic Supplementary Material (ESI) for Soft Matter.
This journal is © The Royal Society of Chemistry 2022

Supporting information: Sensitivity of viscoelastic characterization in intermodulation atomic force microscopy

Abhilash Chandrashekar,[1,*] Arthur Givois,[1,†,*] Pierpaolo Belardinelli,[2] Casper L. Penning,[1] Alejandro M. Aragón,[1] Urs Staufer,[1] and Farbod Alijani[1,‡]

[1]*Faculty of Mechanical, Maritime and Materials Engineering, Delft University of Technology, Mekelweg 2, 2628 CD Delft, The Netherlands*
[2]*DICEA, Polytechnic University of Marche, Ancona, Italy*

S1. EXPERIMENTAL DATA PROCESSING

We measure the spectral components of the cantilever motions d_c in free, lift and engaged motions $(\widetilde{d}_{\text{free}}, \widetilde{d}_{\text{eng}}, \widetilde{d}_{\text{lift}})$, which correspond to tip motions measured at decreasing distances from the sample as described in Fig. S1.1.

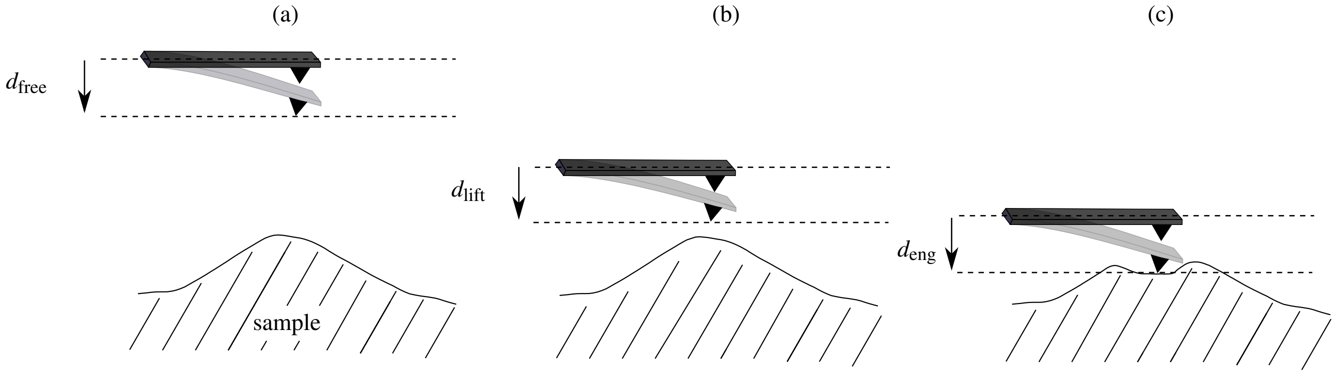

FIG. S1.1: Schematic of the different working positions ((a) free - (b) lift - (c) engaged) for the measurements of the cantilever displacement.

The lift motion denotes the motion of the cantilever at a position close to the surface. It provides a measure to compensate the contribution of long-range linear forces due to squeeze-film damping or electrostatic interactions. These effects are embedded in the linear transfer function of the so-called background forces $\widetilde{\chi}_{\text{BG}}$ [1]. From the measurements of $(\widetilde{d}_{\text{free}}, \widetilde{d}_{\text{eng}}, \widetilde{d}_{\text{lift}})$ we estimate the tip-sample nonlinear force at intermodulation frequencies by applying [2]:

$$\widetilde{F}_{\text{ts}}^{(c)}(\omega) = k\left[-\frac{\omega}{\omega_0}^2 + j\frac{\omega}{\omega_0} + 1\right]\left(\widetilde{d}_{\text{eng}}(\omega) - \widetilde{d}_{\text{free}}(\omega)\right) - \widetilde{\chi}_{\text{BG}}^{-1}\widetilde{d}_{\text{eng}}, \tag{1}$$

in which the last term corresponds to the background force compensation, with its associated linear transfer function defined by

$$\widetilde{\chi}_{\text{BG}}^{-1}(\omega) = k\left[-\frac{\omega}{\omega_0}^2 + j\frac{\omega}{\omega_0} + 1\right]\left(\frac{\widetilde{d}_{\text{lift}} - \widetilde{d}_{\text{free}}}{\widetilde{d}_{\text{lift}}}\right) \tag{2}$$

and approximated on the narrow frequency band with the polynomial [1]:

$$\widetilde{\chi}_{\text{BG}}^{-1}(\omega) \approx k(a\omega^2 + jb\omega). \tag{3}$$

The coefficients a and b come from the fit of Eq. (2) at the two drive frequencies (ω_1, ω_2). In addition, we apply the following phase rotation to compensate the phase shift potentially caused by a time delay inherent to the processing equipment [3]:

$$\widetilde{F}_{\text{ts,exp}}(\omega) = \widetilde{F}_{\text{ts}}^{(c)}(\omega)\mathrm{e}^{-j(R_0 + R_1\omega/\omega_c)} \tag{4}$$

* These two authors contributed equally
† Present address: Université de Technologie de Compiègne, Roberval (Mechanics, Energy and Electricity), Centre de Recherche Royallieu, CS 60319, 60203 Compiègne Cedex, France. E-mail: arthur.givois@utc.fr
‡ E-mail: f.alijani@tudelft.nl

where $\omega_c = \frac{1}{2}(\omega_1 + \omega_2) \approx \omega_0$, and $\widetilde{F}_{\mathrm{ts}}^{(\mathrm{c})}$ denotes the tip-sample intermodulation components with the rotation coefficients (R_0, R_1) adjusted such that $\arg(d_{\mathrm{eng}}(\omega_1)) = \arg(d_{\mathrm{eng}}(\omega_2)) = 0$:

$$R_0 = \arg(d_{\mathrm{eng}}(\omega_1)) - \frac{\arg(d_{\mathrm{eng}}(\omega_2)) - \arg(d_{\mathrm{eng}}(\omega_1))}{\omega_2 - \omega_1}\omega_1 \tag{5}$$

$$R_1 = \frac{\arg(d_{\mathrm{eng}}(\omega_2)) - \arg(d_{\mathrm{eng}}(\omega_1))}{\omega_2 - \omega_1}\omega_c. \tag{6}$$

The phase equalization procedure defined by Eqs. (4)-(6) is also applied on the simulated components for comparison purposes.

S2. ADDITIONAL NUMERICAL DATA

A. Simulations

The driving force signal $F_{\mathrm{d}}(t)$ used in the simulations is defined specifically for the experimental data considered in the study. In particular, it is estimated for the set of frequency, stiffness and quality factor of the first resonance of the cantilever f_0, k and Q obtained from the thermal calibration. The excitation signal is obtained from the free motion frequency components as :

$$F_{\mathrm{d}}(t) = \sum_{\omega\in\omega_{\mathrm{IM}}} 2|\widetilde{F}_{\mathrm{d}}(\omega)|\cos(\omega t + \arg(\widetilde{F}_{\mathrm{d}}(\omega))) \tag{7}$$

with

$$\widetilde{F}_{\mathrm{d}}(\omega) = k\big[-\frac{\omega}{\omega_0}^2 + j\frac{\omega}{Q\omega_0} + 1\big]\widetilde{d}_{\mathrm{free}}(\omega) \tag{8}$$

where the ω_{IM} denotes the pulsation of intermodulation [4].

The time signals are simulated using the following dimensionless values:

$$\overline{d}_c = \frac{d_c}{A}, \quad \overline{d}_s = \frac{d_s}{A}, \quad \overline{F}_d = \frac{F_d}{kA}, \quad \overline{F}_{\mathrm{ts}} = \frac{F_{\mathrm{ts}}}{kA}, \quad \overline{t} = \omega_0 t, \quad \overline{h} = \frac{h}{A}, \quad \overline{s} = \frac{s}{A}. \tag{9}$$

in which the displacement of reference is the amplitude of the engaged motion at the second drive frequency $A = |d_{\mathrm{c}}|_{\omega=\omega_2}$. The following dimensionless design parameters are considered in the numerical procedure:

$$\overline{F}_{\mathrm{ad}} = \frac{F_{\mathrm{ad}}}{kA}, \quad \overline{k}_v = \frac{k_v}{k}, \quad \overline{k}_s = \frac{k_s}{k}, \quad \overline{\eta}_v = \frac{\eta_v\omega_0}{k}, \quad \overline{\eta}_s = \frac{\eta_s\omega_0}{k}. \tag{10}$$

Thus, the equation of motion (Eq.(1) of the main manuscript) is

$$\ddot{d}_{\mathrm{c}} + \frac{\dot{d}_{\mathrm{c}}}{Q} + d_{\mathrm{c}} = F_{\mathrm{d}}(t) + F_{\mathrm{ts}}(s,\dot{s}) \tag{11}$$

in which the overbars are dropped for the sake of brevity. The time signals are computed by simulating Eq. (11) using a Runge-Kutta scheme. At low sample relaxation times $\overline{\tau_s} = \overline{\eta}_s/\overline{k}_s < 10^{-3}$, a scheme designed for stiff systems is employed (the *ode*23*s* function of Matlab is used, instead of the classical *ode*45 time integration solver). The signals for d_c, d_s and F_{ts} are simulated on 8 ms, which corresponds to four intermodulation beatings since $\Delta f = \frac{\omega_2-\omega_1}{2\pi} = 500$ Hz is applied in experiments. A zero initial condition for displacements and velocities is applied.

In order to convert the simulated tip-sample force signal $F_{\mathrm{ts,sim}}$ from time to frequency domain at intermodulation frequencies, we extract two beat periods in steady state oscillations (Fig. S2.1 (a-b)). The amplitude and phase components $(|\widetilde{F}_{\mathrm{ts,sim}}|, \phi_{F_{\mathrm{ts}}})$ of 32 intermodulation frequencies are estimated using a synchronous detection scheme [5]. Next, we use a sliding window with a length equal to one beat period as shown in Fig. S2.1 (c) and take 10 estimations of the phase and amplitude components. The estimations are then averaged to reduce numerical noise as shown in Fig. S2.1(d). Finally, the spectral components of the interaction force are stored in the same way as in experiments, in complex form like $\widetilde{F}_{\mathrm{ts,sim}} = |\widetilde{F}_{\mathrm{ts,sim}}|\mathrm{e}^{j\phi_{F_{\mathrm{ts}}}}$.

The objective function used for estimating the viscoelastic parameters is defined by [4, 6]:

$$f(\boldsymbol{P}) = \sqrt{\sum_{\omega=\omega_{Im}} |\widetilde{F}_{\mathrm{ts,exp}}(\omega) - \widetilde{F}_{\mathrm{ts,sim}}(\omega,\boldsymbol{P})|^2}. \tag{12}$$

In order to minimize Eq. (12) we use Levenberg-Marquardt algorithm [7] and combine it with nonlinear least squares (*lsqnonlin* function) in Matlab. This least-square minimization is performed with an iterative procedure which involves the computation of the partial derivatives (gradient) at each iteration. A parallel implementation on a small cluster was used to perform multiple minimization routines: approximately 10 nodes and 36 hours in total were needed to obtain the results shown in Fig. 5 of the main manuscript. We show in table S2.1 the lower and upper limit of parameter values defined for the optimization. These parameter ranges are deliberately wide because we assume that we have no prior knowledge of the material properties, except in the case of the probe height for which a first approximation can be extracted from the force quadrature curves (see section S2.C).

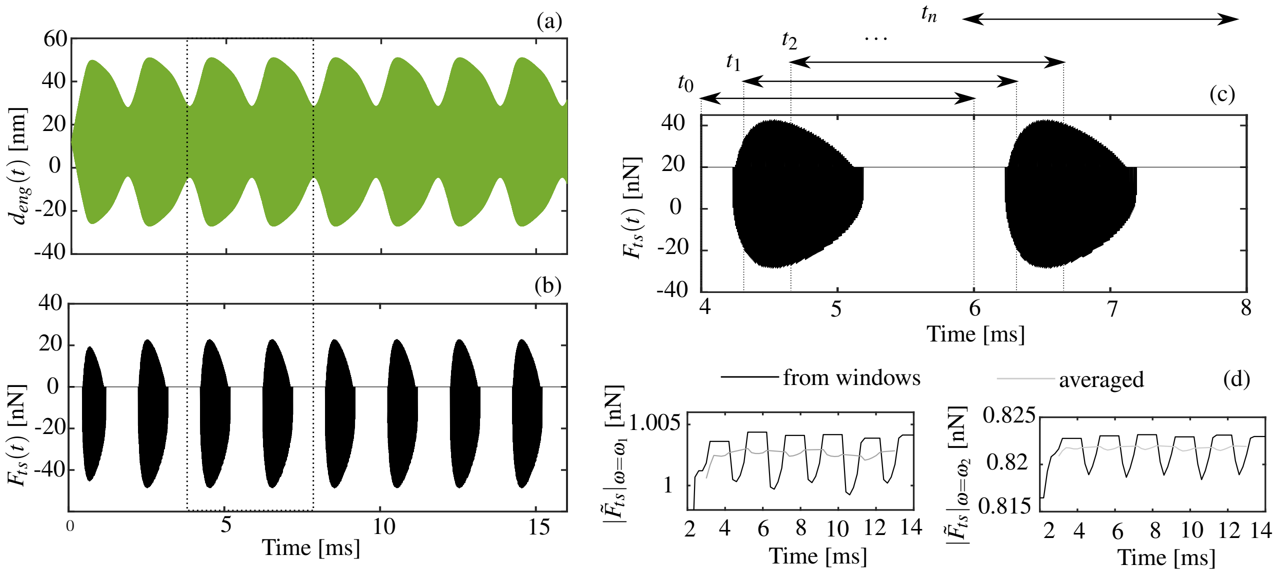

FIG. S2.1: Example of simulated tip displacement (a) and tip-sample force (b) signals. Zoom on the extracted portion of the force signal used for the estimation of the observables (c). Comparison between the direct and averaged amplitudes of the interaction force at the two drive frequencies ω_1 and ω_2 (d).

Parameter	F_{ad} [nN]	k_v [N.m^{-1}]	η_v [mg.s^{-1}]	k_s [N.m^{-1}]	η_s [mg.s^{-1}]	h [nm]
Minimum value	0.05	≈ 0	≈ 0	≈ 0	≈ 0	5
Maximal value	100	$10k$	$10k/\omega_0$	$20k$	$20k/\omega_0$	45

TABLE S2.1: Parameter ranges used for the optimization routine. Here, k represents the cantilever stiffness in N/m and ω_0 represents the first resonance frequency in rad/s

B. Results of global optimization tests on synthetic data

In this section we discuss the use of a global optimization procedure for parameter estimation and further elaborate on the limitations of the procedure. In general, global optimization techniques such as Particle swarm optimization do not rely on gradient descent method used by local optimization techniques like the Levengerg-Marquardt method, and hence don't require a differentiable objective function. Such a characteristic helps to determine if the lack of sensitivity of surface motion can be attributed to the chosen optimization algorithm or it is linked to model parameters. Additionally, a global optimization method has the advantage that a large parameter space can be searched from different initial starting points without having prior knowledge on the optimum solution. However, in order to obtain a physically interpretable solution and to reduce the computational time, it is necessary to restrict the search range. We achieve this by assigning values for each of the model parameter from previous experimental characterizations and then extending their ranges by an order of magnitude[8–10].

In particular, we choose the sample parameters suitable for PS-LDPE material and generate synthetic data sets based on the interaction with a Silicon cantilever. The sample properties used for the simulations is provided in table S2.2 together with the following cantilever properties: f_0 = 163 kHz, Q = 491, k = 23.95 N m^{-1}, the effective driving force F_d = 1.39 nN and the unperturbed height h = 22.6 nm. Next, we use random sampling to select different starting parameter sets. A total of 15 different parameter sets are created and simulated with the moving surface model to generate the amplitude and phase frequency components which are then used as references (i.e. parameter sets for which the objective function is zero) for the Particle swarm based global optimization.

Table S2.2 shows the optimization results for 4 randomly chosen parameter sets out of 15 simulated data sets. The results show that tip-sample dynamics is well approximated with low error values E, but the identified parameter values are far from their true values. This deviation is far more significant for surface parameters in comparison with bulk parameters. Once again, we attribute this issue to non-convexity and lack of sensitivity of surface parameters as discussed in the main manuscript. Additionally, Figs. S2.2 and S2.3 show the time trace of the cantilever and the associated surface motion together with the force quadratures for both the original dynamics coming from the model simulations and the identified dynamics resulting from optimization. In both the figures, while we observe a good agreement for the force quadratures and cantilever motion, the identified motion of the sample surface does not match with the simulated motion (See Figs S2.2(g)-(h) and S2.3(g)-(h)). This further confirms the trivial contribution of the surface motion on amplitude and phase of intermodulation components.

Parameter Set	Designation	F_{ad} [nN]	k_v [N.m^{-1}]	η_v [mg.s^{-1}]	k_s [N.m^{-1}]	η_s [mg.s^{-1}]	E (nN)
P_1	Optimum	2.98	2.60	0.199	8.31	0.0371	$3.80 \cdot 10^{-3}$
	PSO result	2.49	2.04	0.181	81.0	2.14	
	Error	16.4 %	21.3 %	9.17 %	875 %	5.67 $\cdot 10^3$%	
P_2	Optimum	0.161	0.0101	0.141	0.220	1.51	$2.54 \cdot 10^{-4}$
	PSO result	0.165	0.0100	0.135	16.8	0.00155	
	Error	3.00 %	0.547 %	4.05 %	7.53 $\cdot 10^3$%	99.9 %	
P_3	Optimum	4.49	6.81	0.0221	0.108	0.582	$2.11 \cdot 10^{-2}$
	PSO result	8.18	0.97	0.378	0.938	0.00105	
	Error	39.6 %	25.7 %	20.0 %	680 %	99.0%	
P_4	Optimum	0.473	0.349	0.469	65.5	0.0105	$8.12 \cdot 10^{-4}$
	PSO result	0.277	0.283	0.802	1.20	0.0360	
	Error	41.5 %	19.0 %	71.1 %	98.2 %	245 %	

TABLE S2.2: Parameter Convergence for data sets P_1, P_2, P_3 and P_4. Cantilever properties used: $f_0 = 163$ kHz, $Q = 491$, $k = 23.95$ N/m. Scanning properties: $F_d = 1.39$ nN, $h = 22.6$ nm, and 41 amplitude and phase intermodulation products. PSO denotes the Particle Swarm Optimization method.

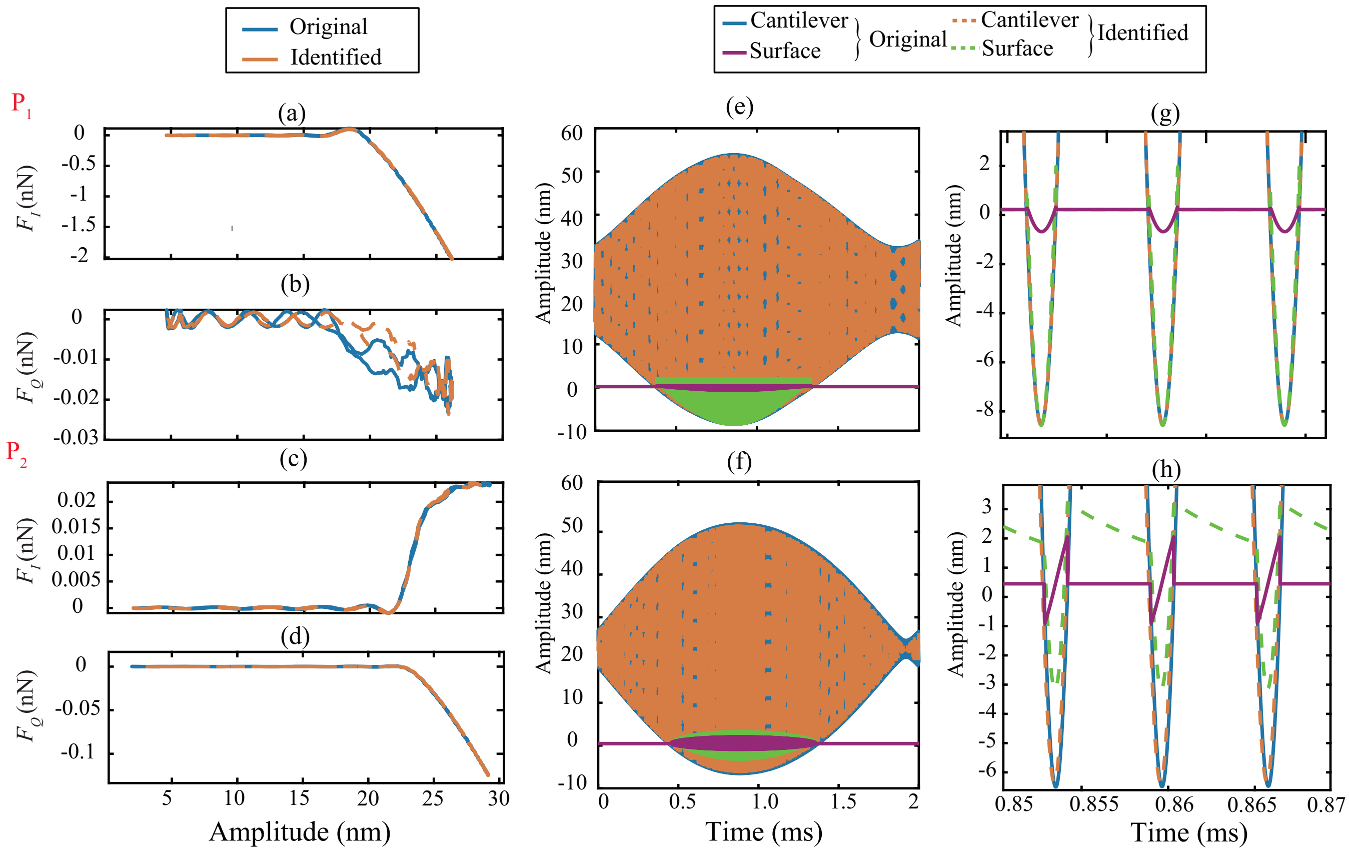

FIG. S2.2: Global optimization results for parameter sets 1 and 2. Cantilever properties: $f_0 = 163$ kHz, $Q = 491$, $k = 23.95$ N/m. Scanning properties: $F_d = 1.39$ nN, $h = 22.6$ nm, and 41 amplitude and phase intermodulation products. (a)-(d) Force quadratures showing the conservative and dissipative tip-sample interactions. The blue color represents the original quadratures obtained from model simulations and the orange color represents the identified quadratures based on optimization. (e)-(f) Time data depicting the motion of the cantilever and the corresponding surface motion due to tip-sample interaction. right: (g)-(h) Zoomed surface motion indicating discrepancies between the original and the identified surface dynamics. The blue and purple color represents the original cantilever and surface dynamics obtained from model simulations; whereas, the orange and green color the original cantilever and surface dynamics based on optimization.

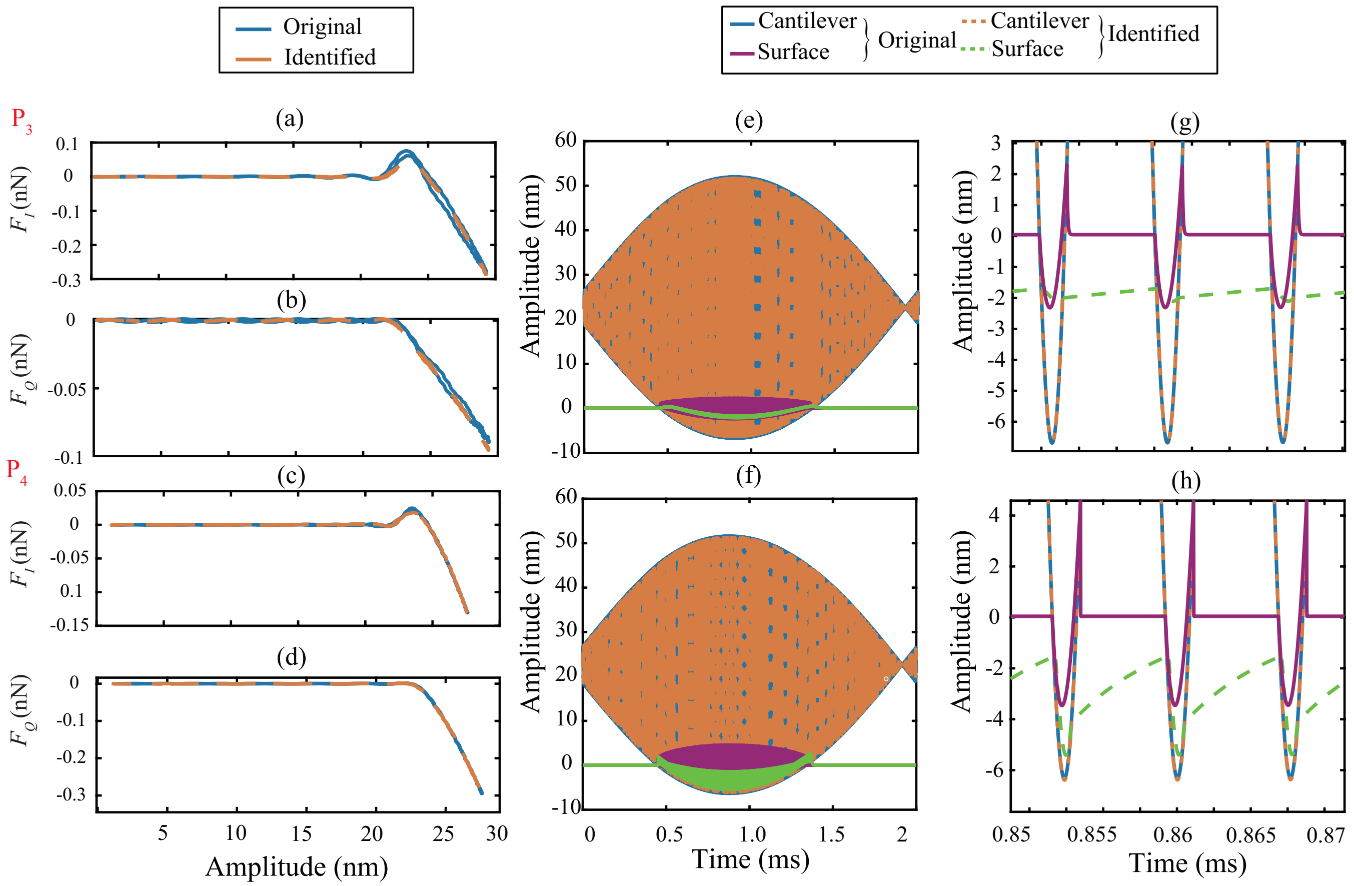

FIG. S2.3: Global optimization results for parameter sets 3 and 4. Cantilever properties: $f_0 = 163$ kHz, $Q = 491$, $k = 23.95$ N/m. Scanning properties: $F_d = 1.39$ nN, $h = 22.6$ nm, and 41 amplitude and phase intermodulation products. (a)-(d) Force quadratures showing the conservative and dissipative tip-sample interactions. The blue color represents the original quadratures obtained from model simulations and the orange color represents the identified quadratures based on optimization. (e)-(f) Time data depicting the motion of the cantilever and the corresponding surface motion due to tip-sample interaction. right: (g)-(h) Zoomed surface motion indicating discrepancies between the original and the identified surface dynamics. The blue and purple color represents the original cantilever and surface dynamics obtained from model simulations; whereas, the orange and green color the original cantilever and surface dynamics based on optimization.

C. Criterion for probe height identification from force quadratures

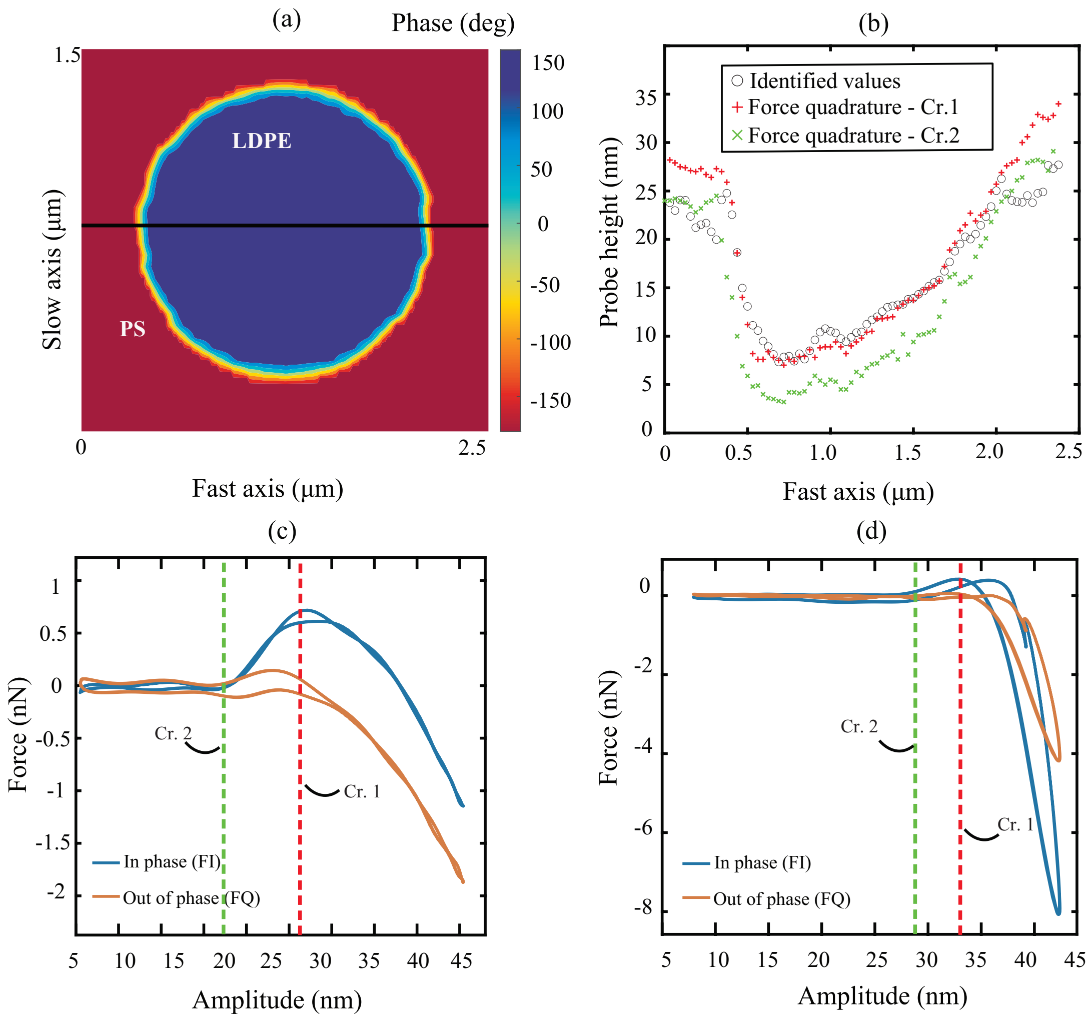

FIG. S2.4: Portion of the extracted line for the analysis in the AFM image (top left). Identified h and reported values directly read from the force quadratures (top right). Illustration of the two criteria (dashed lines) for estimating the probe height on the quadratures on one pixel made of PS (bottom left) and of LDPE (bottom right).

The probe height h is included in the set of unknown parameters (see main manuscript modeling section III). In general h varies with the working height of the cantilever which in turn depends on how much the feedback control moves the z-piezo during the scanning operation. By taking advantage of the conservative quadrature, in phase with the cantilever motion, it is possible to estimate an approximate value for h based on the onset of repulsive forces.

We suggest two criteria for extracting h from force quadratures as illustrated in Fig. S2.4. We assume the maximum of the in-phase force component (related to adhesion) is achieved closely after the tip starts to penetrate the sample. Thus, the first criterion (denoted by red crosses in Fig. S2.4 (b)) is taken at the middle of the increasing part of F_I, whereas the second one corresponds to the amplitude where the in-phase component starts to increase. We browse and apply these two criterion on all pixels of the black line displayed in Fig. S2.4(a). The comparison shown in Fig. S2.4(b) highlights a better match between the heights corresponding to LDPE pixels using the first criteria, when the second criteria seems more suited for the pixels related to PS material. That can be explained by the different material properties, for instance the larger stiffness for PS causes a faster increase of F_I, whereas in case of the softer material the short-range adhesive force is more significantly involved before the tip starts to indent the sample. The analysis of these force quadrature curves could be further developed using a more accurate tip-sample force model such as Attard's model [11–15], in order to describe first the transition between the non-contact and adhesive regime, and secondly the transition between the adhesive and repulsive regime.

S3. HIGH VOLUME GRADIENT BASED OPTIMIZATION AND INITIAL POINT SELECTION PROCEDURE

In this section, we discuss the results obtained using the Levenberg-Marquardt algorithm from multiple initial points for both models with and without sample's surface motion. This is done to analyze the sensitivity of the model on initial starting points for the optimization. We begin by creating a numerical range for each parameter based on previous literature studies. Then, a grid of initial starting points is chosen and for each initial point we perform the optimization routine. The distribution of the identified parameters is analysed with histograms and by fitting Gaussian function to extract statistics. The distribution are discussed for each model separately in the following sections.

A. Piecewise linear model with surface motion

Using the moving surface model, we run multiple gradient-based optimizations for pixel (i) and pixel (iii) of Fig. 2 in the main manuscript with the grid of initial parameters defined in table S3.3. The grid includes 3 different values per parameters, chosen in such a way that the parameter exploration recovers a large parameter space (including notably at least one order of magnitude in the case of the viscoelastic properties), and that all routines are performed within a reasonable computational time. In total, $3^6 = 729$ optimizations were performed, starting from all the combinations of the grid. In this section we present the histograms used to extract the values reported in table I of the main manuscript.

F_{ad} [nN]	k_v [N.m^{-1}]	η_v [mg.s^{-1}]	k_s [N.m^{-1}]	η_s [mg.s^{-1}]	h [nm]
[5 25 45]	[0.02 1 40]	[0.2 1 5]	[0.02 1 40]	[0.2 1 5]	[15 25 35]

TABLE S3.3: Grid of initial points for the local optimization procedure using the moving surface model.

Figures S3.5 and S3.6 highlight the distribution of the identified parameters with respect to the objective function for pixels (i) and (iii), respectively. We see a clear correlation between a large distribution and low errors only for some parameters such as $F_a d$, k_v, η_v, h for pixel (iii) in Fig. S3.5. If model parameters have strong correlation with the objective function then the maximum of the histogram counts (rows 1 and 3) coincides with the minima of the scatter plots (rows 2 and 4). For example, in case of Figs. S3.5(a) and (d), we look at the influence of adhesion force $F_a d$ on the objective function and we observe that the location of the maximum along the x-axis in Fig. S3.5(a) coincides with the minima along the same x-axis in Fig. S3.5(d). A similar behaviour is observed in Figs. S3.5 (b)&(e), (c)&(f), and(i)&(l). On the contrary, Figs. S3.5 (g)&(j) and (h)&(k) lack such property and instead exhibit random and spread distributions. This behavior is due to the insensitivity of the objective function to the sample parameters. A similar observation holds for the PS material (Fig. S3.6) with an even more complex distribution. It is here attributed to the combined effect of non-convexity and insensitive regions in which the optimizer encounters a stopping condition.

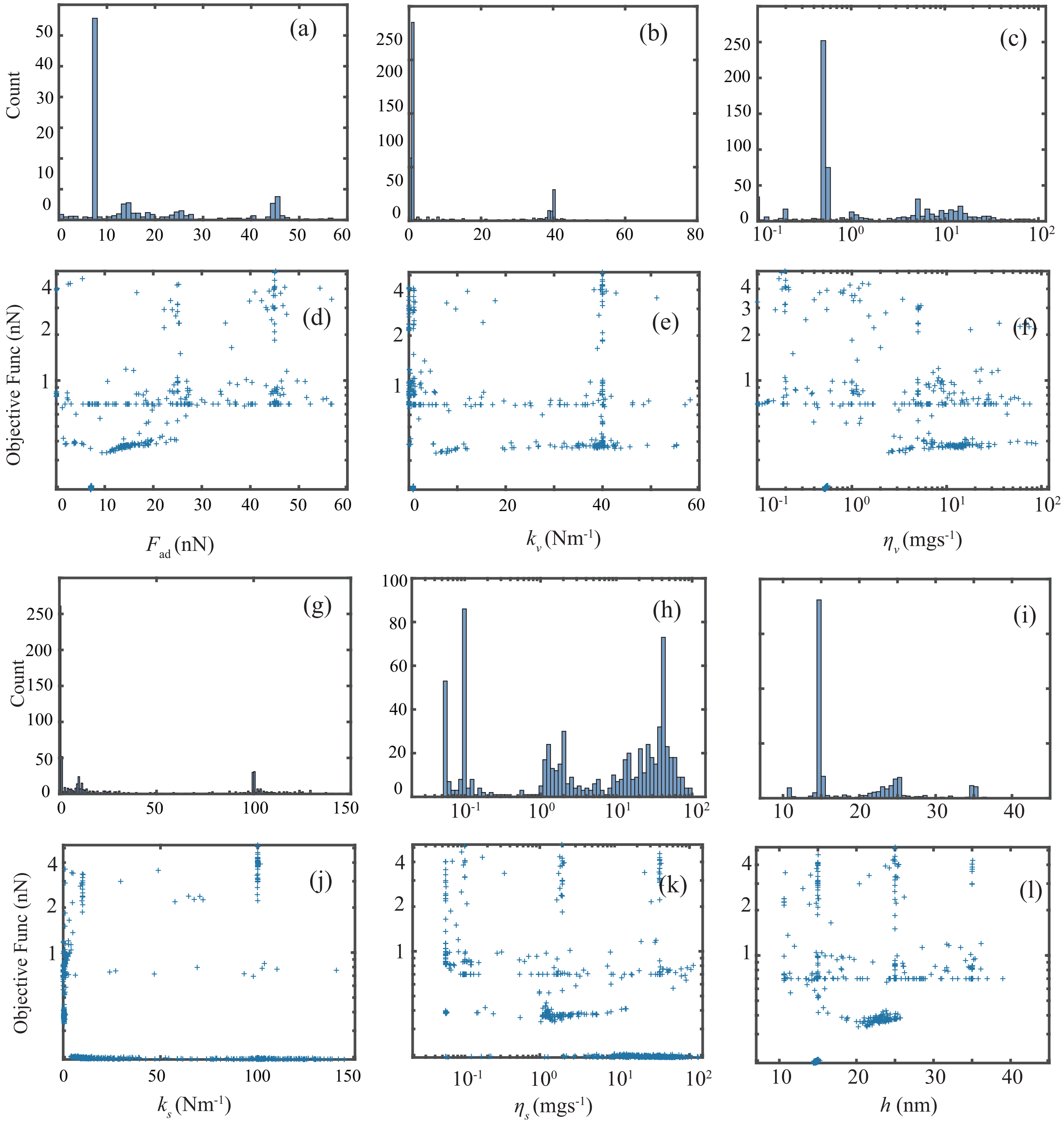

FIG. S3.5: Identified parameters of the PWL model with sample motion, obtained on LDPE material at pixel (iii) of Fig. 2(b) in the main manuscript with the initial positions defined in table S3.3. Parameter distributions and errors are respectively plotted in (a)&(d) for F_ad, (b)&(e) for k_v, (c)&(f) for η_v, (g)&(j) for k_s, (h)&(k) for η_v and (i)&(l) for h.

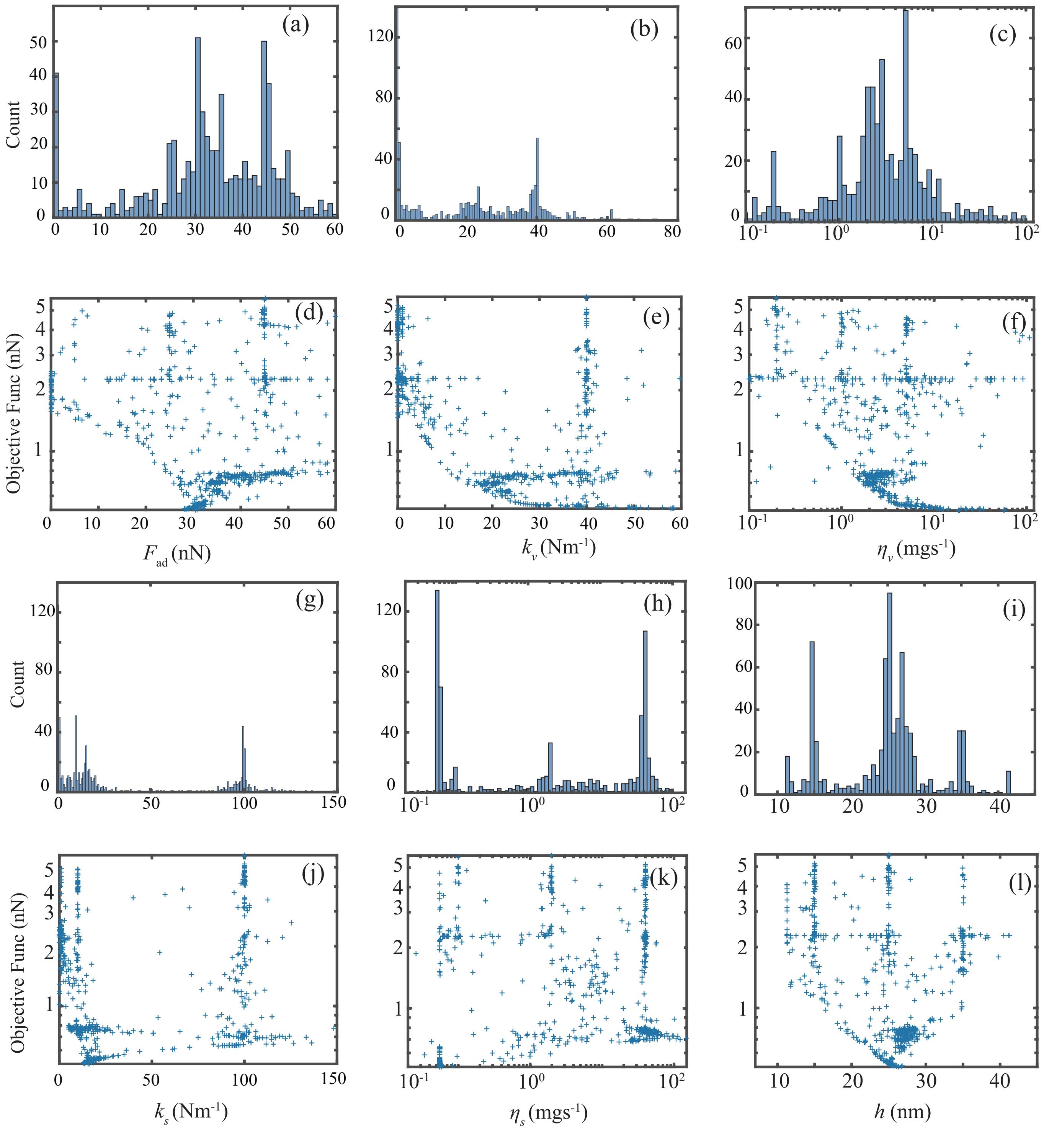

FIG. S3.6: Identified parameters of the PWL model with sample motion, obtained on PS material at pixel (i) of Fig. 2(b) in the main manuscript with the initial positions defined in table S3.3. Parameter distributions and errors are respectively plotted in (a)&(d) for F_ad, (b)&(e) for k_v, (c)&(f) for η_v, (g)&(j) for k_s, (h)&(k) for η_v and (i)&(l) for h.

B. Piecewise linear model without surface motion

Here, we report the results and histograms obtained from the large set of optimizations carried out using the piecewise linear model without surface motion. We begin with a set of 3^4 initial parameters defined by the grid presented in table S3.4, and analyze the parameter distributions in the same way as outlined in the previous section.

With the 4 parameters model, statistic for the identified parameters depicts well defined Gaussian distributions that are specific for each type of material. Additionally, the mean of the Gaussian distributions correspond to the lowest values of the objective function. This is shown in Figs. S3.5 and S3.6 for PS and LDPE material sampled at pixel locations (i) and (iii) of Fig. 2 in the main manuscript. The parameter values from the optimization procedure are reported in table S3.5.

F_{ad} [nN]	k_v [N.m^{-1}]	η_v [mg.s^{-1}]	h [nm]
[5 25 45]	[0.02 1 40]	[0.2 1 5]	[15 25 35]

TABLE S3.4: Grid of initial points for the local optimization procedure using PWL model without sample motion.

	Pixel (i)	Pixel (iii)
F_{ad} [nN]	32.7 ± 0.45	7.13 ± 0.008
k_v [N/m]	17.52 ± 0.52	0.854 ± 0.002
η_v [mg/s]	1.975 ± 0.006	0.519 ± 0.001
h [nm]	26.7 ± 0.18	14.7 ± 0.03

TABLE S3.5: Identified parameters resulting from the Gaussian fits. We extracted the results with errors smaller than 0.71 nN for pixel (i) (cf Fig. S3.8) and 0.25 nN for pixel (iii) (cf Fig. S3.7). The uncertainties are estimated with a 95% confidence interval.

From this statistical analysis, we extract a reduced set of starting parameters. The parameters summarised in table S3.6 have been used to obtain the results showcased in Fig. 5 of the main manuscript. The two first initial points in Table S3.6 were selected by identifying the mean values (also corresponding with the lowest error) among the final results displayed in Figs. S3.7 and S3.8. In addition, we add a third initial point leading to identified parameters within the confidence intervals for all parameters and both the pixels. We detail the final parameters and errors obtained on pixels (i) and (iii) with these three initial points in table S3.7.

F_{ad}	k_v	η_v	h
[nN]	[N/m]	[mg/s]	[nm]
45	0.02	0.2	35
5	1	1	35
5	1	1	15

TABLE S3.6: Initial starting parameters used as inputs for the optimization performed on the AFM scan.

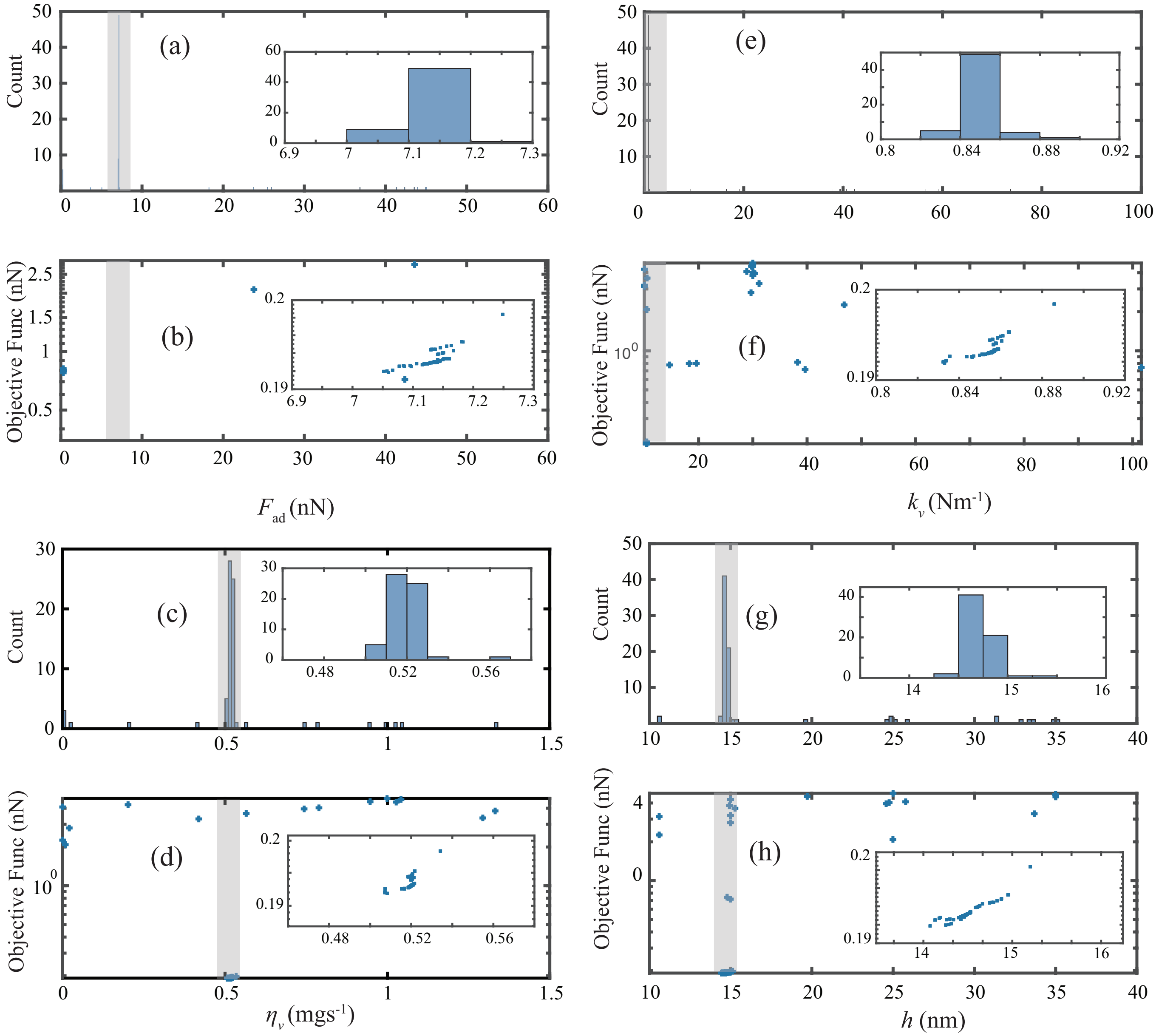

FIG. S3.7: Identified parameters of the PWL model without sample motion, obtained on pixel (iii) of Fig. 2(b) in the main manuscript (LDPE) from the initial positions defined in table S3.4. Parameter distributions and errors are respectively plotted in (a)&(b) for F_a, (e)&(f) for k_v, (c)&(d) for η_v and (g)&(h) for h. The shadowed areas highlight the Gaussian distributions.

				Pixel (i)					Pixel (iii)				
Initial parameters				Final parameters				Final Error	Final parameters				Final Error
F_{ad}	k_v	η_v	h	F_{ad}	k_v	η_v	h	E	F_{ad}	k_v	η_v	h	E
[nN]	[N/m]	[mg/s]	[nm]	[nN]	[N/m]	[mg/s]	[nm]	[nN]	[nN]	[N/m]	[mg/s]	[nm]	[nN]
45	0.02	0.2	35	32.9	17.74	1.99	26.9	0.68	0.3	9.33	21.1	31.5	0.78
5	1	1	35	33.8	18.6	2.08	27.0	0.69	7.06	0.833	0.508	14.4	0.192
5	1	1	15	32.0	16.7	1.89	26.6	0.69	7.12	0.852	0.519	14.6	0.193

TABLE S3.7: Identified parameters and final errors obtained at pixels (i) and (iii) from the three selected initial points defined in Table S3.6.

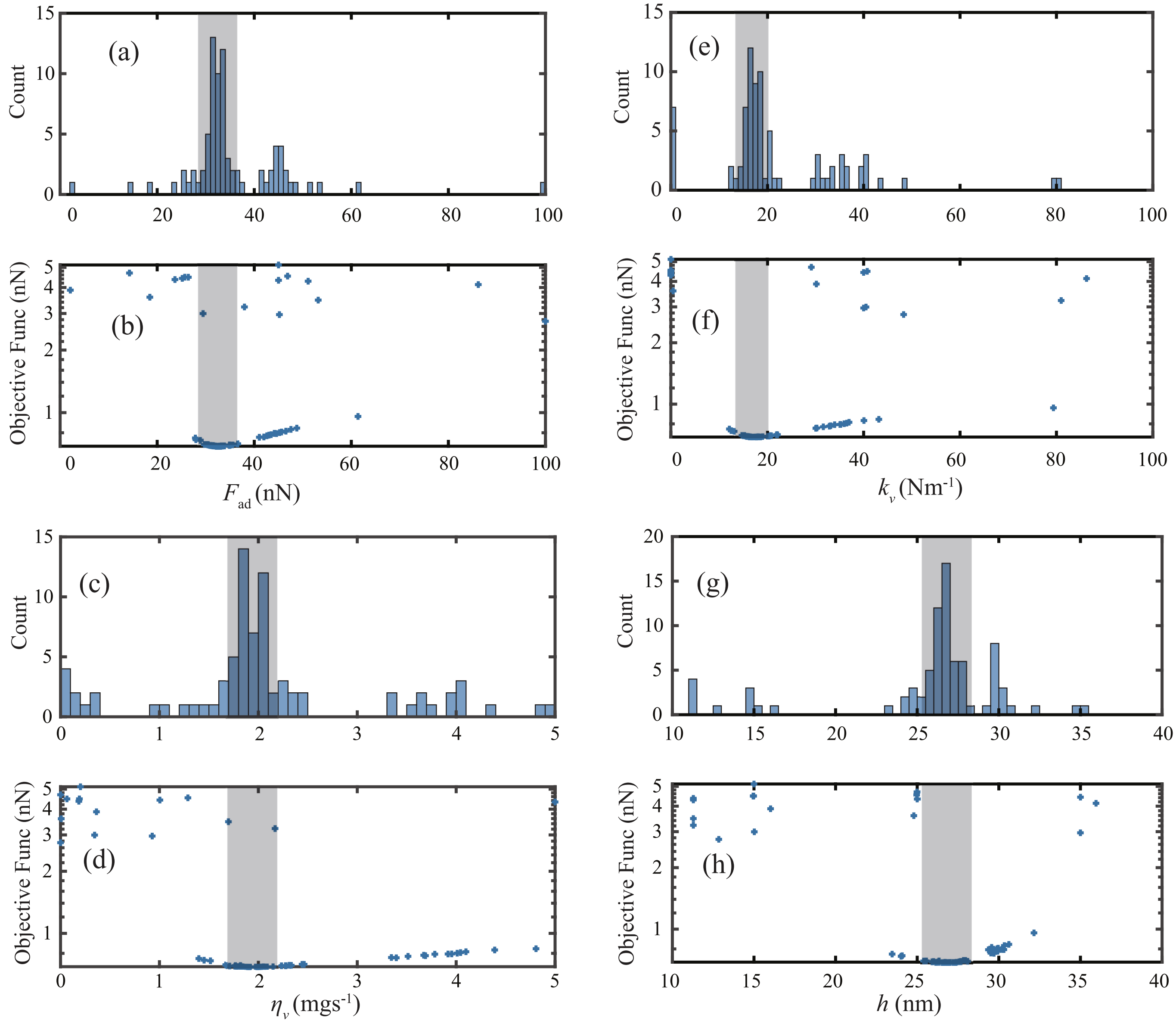

FIG. S3.8: Identified parameters of the PWL model without sample motion, obtained on pixel (i) of Fig. 2 in the main manuscript (PS), starting from the initial positions defined in table S3.4. Parameter distributions and errors are respectively plotted in (a)(b) for F_a, (e)(f) for k_v, (c)(d) for η_v and (g)(h) for h. The shadowed areas highlight the Gaussian distributions.

[1] R. Borgani, P.-A. Thorén, D. Forchheimer, I. Dobryden, S. M. Sah, P. M. Claesson, and D. B. Haviland, Background-force compensation in dynamic atomic force microscopy, Physical Review Applied **7**, 064018 (2017).

[2] C. Hutter, D. Platz, E. Tholén, T. Hansson, and D. Haviland, Reconstructing nonlinearities with intermodulation spectroscopy, Physical review letters **104**, 050801 (2010).

[3] D. Platz, *Reconstructing force from harmonic motion*, Ph.D. thesis, KTH Royal Institute of Technology (2013).

[4] D. Platz, D. Forchheimer, E. A. Tholén, and D. B. Haviland, Interpreting motion and force for narrow-band intermodulation atomic force microscopy, Beilstein journal of nanotechnology **4**, 45 (2013).

[5] E. A. Tholén, D. Platz, D. Forchheimer, V. Schuler, M. O. Tholén, C. Hutter, and D. B. Haviland, Note: The intermodulation lockin analyzer, Review of Scientific Instruments **82**, 026109 (2011).

[6] D. Forchheimer, D. Platz, E. A. Tholén, and D. B. Haviland, Model-based extraction of material properties in multifrequency atomic force microscopy, Physical Review B **85**, 195449 (2012).

[7] K. Levenberg, A method for the solution of certain non-linear problems in least squares, Quarterly of applied mathematics **2**, 164 (1944).

[8] P.-A. Thorén, R. Borgani, D. Forchheimer, I. Dobryden, P. M. Claesson, H. G. Kassa, P. Leclère, Y. Wang, H. M. Jaeger, and D. B. Haviland, Modeling and measuring viscoelasticity with dynamic atomic force microscopy, Phys. Rev. Applied **10**, 024017 (2018).

[9] D. B. Haviland, C. A. van Eysden, D. Forchheimer, D. Platz, H. G. Kassa, and P. Leclère, Probing viscoelastic response of soft material surfaces at the nanoscale, Soft Matter **12**, 619 (2015).

[10] C. L. Penning, Modelling of viscoelasticity using multifrequency afm (2020).

[11] P. Attard, Interaction and deformation of viscoelastic particles. 2. adhesive particles, Langmuir **17**, 4322 (2001).

[12] P. Attard, Measurement and interpretation of elastic and viscoelastic properties with the atomic force microscope, Journal of Physics: Condensed Matter **19**, 473201 (2007).

[13] B. Rajabifar, J. M. Jadhav, D. Kiracofe, G. F. Meyers, and A. Raman, Dynamic afm on viscoelastic polymer samples with surface forces, Macromolecules **51**, 9649 (2018).

[14] B. Rajabifar, A. K. Bajaj, R. G. Reifenberger, R. Proksch, and A. Raman, Discrimination of adhesion and viscoelasticity from nanoscale maps of polymer surfaces using bimodal atomic force microscopy, Nanoscale (2021).

[15] B. Rajabifar, R. Wagner, and A. Raman, A fast first-principles approach to model atomic force microscopy on soft, adhesive, and viscoelastic surfaces, Materials Research Express (2021).
